# Decoding frontotemporal and cell-type-specific vulnerabilities to neuropsychiatric disorders and psychoactive drugs

**DOI:** 10.1098/rsob.240063

**Published:** 2024-06-12

**Authors:** Jiatong Ji, Honglu Chao, Huimei Chen, Jun Liao, Wenqian Shi, Yangfan Ye, Tian Wang, Yongping You, Ning Liu, Jing Ji, Enrico Petretto

**Affiliations:** ^1^ Institute for Big Data and Artificial Intelligence in Medicine, School of Science, China Pharmaceutical University (CPU), Nanjing, Jiangsu 211198, People's Republic of China; ^2^ Department of Neurosurgery, the First Affiliated Hospital of Nanjing Medical University, Nanjing, Jiangsu 210029, People's Republic of China; ^3^ Duke-NUS Medical School, Singapore 169857, Singapore; ^4^ High Performance Computing Center, School of Science, China Pharmaceutical University (CPU), Nanjing, Jiangsu 211198, People's Republic of China; ^5^ Department of Neurosurgery, The Affiliated Kizilsu Kirghiz Autonomous Prefecture People’s Hospital of Nanjing Medical University, Xinjiang, Artux 845350, People's Republic of China; ^6^ Gusu School, Nanjing Medical University, Suzhou, Jiangsu 215006, People's Republic of China

**Keywords:** frontal lobe, temporal lobe, psychiatric disorder, single-cell RNA sequencing, psychoactive drugs

## Abstract

Frontotemporal lobe abnormalities are linked to neuropsychiatric disorders and cognition, but the role of cellular heterogeneity between temporal lobe (TL) and frontal lobe (FL) in the vulnerability to genetic risk factors remains to be elucidated. We integrated single-nucleus transcriptome analysis in ‘fresh’ human FL and TL with genetic susceptibility, gene dysregulation in neuropsychiatric disease and psychoactive drug response data. We show how intrinsic differences between TL and FL contribute to the vulnerability of specific cell types to both genetic risk factors and psychoactive drugs. Neuronal populations, specifically PVALB neurons, were most highly vulnerable to genetic risk factors for psychiatric disease. These psychiatric disease-associated genes were mostly upregulated in the TL, and dysregulated in the brain of patients with obsessive-compulsive disorder, bipolar disorder and schizophrenia. Among these genes, GRIN2A and SLC12A5, implicated in schizophrenia and bipolar disorder, were significantly upregulated in TL PVALB neurons and in psychiatric disease patients’ brain. PVALB neurons from the TL were twofold more vulnerable to psychoactive drugs than to genetic risk factors, showing the influence and specificity of frontotemporal lobe differences on cell vulnerabilities. These studies provide a cell type resolved map of the impact of brain regional differences on cell type vulnerabilities in neuropsychiatric disorders.

## 1. Introduction

Genome-wide association studies (GWAS) elucidated the genetic aetiology and pathophysiology of neuropsychiatric disorders [1], identifying a multitude of associated loci and disease risk factors (aka genetic susceptibility factors), and revealing substantial genetic complexity [2,3]. Several clinical studies shown that diverse neuropsychiatric disorders share similar symptoms, which, at least in part, could be due to common genetic factors underlying these disorders [4], which impact central functional processes such as neuronal regeneration, myelination, synapse maturation and plasticity [5,6].

In addition to the role played by genetics, the frontal lobe (FL) and temporal lobe (TL) are believed to be involved in the pathophysiology of neuropsychiatric disorders, including bipolar disorder (BP), schizophrenia (SCHI) and autism spectrum disorder (ASD) [7–9]. For example, changes in cortical volume, surface area and thickness have been reported in in schizophrenia patients, who show significant reduction of the entire inferior temporal gyrus, while bipolar patients showed significant reduction of cortical thickness in the main TL as well as part of the FL and parietal lobe [10]. Schizophrenic patients also exhibit functional, structural and metabolic abnormalities in the prefrontal cortex (PFC) [11]. Other studies showed structural alterations in the hippocampus and in medial TL regions in patients with schizophrenia, bipolar disorder and depression [12]. However, most of these studies focussed on structural brain changes between FL and TL in patients with psychiatric disorders, and a link between these structural changes and dysregulation of specific cellular processes and/or cell vulnerabilities in disease has not firmly established yet.

The regional differences between TL and FL might also affect gene expression in specific cell types, and, in turn, these might impact important functional processes dysregulated in neuropsychiatric disorders. For example, region-specific characteristics of neurons in the cortex, including different electrophysiological features, have been previously reported [13], and region-specific transcriptomic changes in neurons from the cerebral cortex (and the subcortical region) showed specific patterns of molecular and developmental regulation [13]. Cellular level differences between brain regions might also be relevant to disease pathobiology; e.g. excitatory neurons (EX) and inhibitory neurons (INH) in the middle frontal gyrus showed a significant association with neuropsychiatric disorders [14], and further cell type and brain area associations have been reported for depressive disorder [15]. In ASD patients, the expression level of synaptic and neurodevelopmental genes in layer 2/3 cortical neurons from PFC is especially affected [16]. However, given the very high cellular complexity within and between brain regions such as TL and FL, cell type-specific and regional-specific gene expression changes remain to be fully elucidated.

Single-cell/nuclei sequencing approaches now permit to analyse and ‘chart’ disease-associated gene expression changes at the cellular level. For example, specific cell types associated with schizophrenia and anorexia nervosa have been identified by integrating mouse single-nucleus RNA-sequencing (snRNA-seq) of brain with GWAS data [17]. However, the relationship between the vulnerable cell type in psychiatric disorders and the cellular processes involved, especially the frontotemporal cortex, is less well-studied and remains poorly understood. Previous studies integrated snRNA-seq analysis with existing drug-specific signatures to determine cell type-specific vulnerabilities [18], but the relative contribution of genetic risk factors and psychoactive drugs to cell type vulnerability, and the effect for brain regional differences, have not been characterized. To bridge this knowledge gap, here we integrated snRNA-seq data from fresh brain tissue samples of TL and FL, with (i) GWAS data for seven psychiatric disorders, (ii) RNA-seq data from healthy and diseased human brain cortex, and (iii) drug-specific gene targets for common psychoactive drugs. This allowed us to define a detailed map of single-cell changes associated with brain regional differences (TL and FL) and assess how these affect cell type vulnerability to both psychiatric genetic risk factors and psychoactive drugs. We also linked changes in TL and FL cell types to gene dysregulation in human brain tissue from patients with neuropsychiatric disorders. To replicate our main findings, we also analyzed single nuclei data from the Allen Brain Atlas. The integrated datasets and results presented here provide a unique resource to help understanding the cell-specific changes during disease pathogenesis and pharmacological treatments using common psychoactive drugs.

## 2. Methods

### 2.1. Tissue collection and library preparation

Three human brain samples were obtained from patients without any psychiatric disorder history in the First Affiliated Hospital of Nanjing Medical University, which was approved by the Research Ethics Committee of Nanjing Medical University (Nanjing, Jiangsu, China), and experiments were performed in accordance with the approved guidelines. The detailed information and the sampling site are found in electronic supplementary materials, table S3 and figure S13, respectively. Three normal tissues were obtained from the healthy part during surgery of multiple intracranial small space-occupying diseases. When selecting the sampling site, we used preoperative MRI images to select a grey matter of the cerebral cortex that was far away from the primary lesion and oedema. Liquid nitrogen is used to inhibit the tissue from degradation. All the experiments were performed in accordance with the approved guidelines.

The snRNA-seq analysis was performed by NovelBio Co., Ltd. The tissues were surgically removed and snap-frozen in liquid nitrogen for intact nucleus isolation. The nucleus isolation was carried out by Nuclei Isolation Kit: Nuclei EZ Prep (NUC101; Sigma, St. Louis, MO). Briefly, the frozen tissue was homogenized in NLB buffer which contain 250 mM sucrose, 10 mM Tris–HCl, 3 mM MgAc2, 0.1% Triton X-100 (Sigma-Aldrich, St Louis, MO, USA), 0.1 mM EDTA, 0.2 U μl^−1^ RNase Inhibitor (Takara, Shiga, Japan). Various concentrations of sucrose were used to purify the nucleus. The concentration of the nucleus was adjusted to about 1000 nuclei/μl for snRNA-Seq. The snRNA-Seq libraries were generated using the 10× Genomics Chromium Controller Instrument and Chromium Single Cell 3’V3.1 Reagent Kits (10× Genomics, Pleasanton, CA, USA). 10× Genomics uses a microfluidic system for cell sorting. Cells and enzymes, combined with Gel Beads, enter the oil phase to form GEMs. The resulting sample libraries were sequenced on separate lanes. To enhance sequencing depth, the primary target number of nuclei for the two samples from TL is set at 10 000, considering an RNA integrity number (RIN) of 6.5. In contrast, for the sample from FL, the target is set at 20 000 nuclei due to a higher RIN of 8.1. Briefly, cells nuclei were concentrated to 1000 nuclei/μl then loaded into each channel to generate single-cell Gel Bead-In-Emulsions (GEMs). After the RT step, GEMs were broken and barcode-cDNA was purified and amplified. The amplified barcoded cDNA was fragmented, A-tailed, ligated with adaptors and index PCR amplified. The final libraries were quantified using the Qubit High Sensitivity DNA assay (Thermo Fisher Scientific, Waltham, MA, USA) and the size distribution of the libraries were determined using a High Sensitivity DNA chip on a Bioanalyzer 2200 (Agilent, Santa Clara, CA, USA). All libraries were sequenced by Novaseq6000 (Illumina, San Diego, CA, USA) on a 150 bp paired-end run.

### 2.2. Preprocessing of single-cell RNA-sequencing data

Raw reads were processed using the CellRanger software (v. 6.0.3). The ‘cellranger count’ command was used to generate the gene-per-cell expression matrices for each sample by aligning the reads to the genome and quantifying expression of the reference genes (GRCh38). Three single-cell gene expression matrices corresponding to the three biological samples were generated. SnRNA-seq data were mainly processed by R package Seurat (v. 4.0.2) [19]. The expression matrices of the three samples were merged into a global Seurat object using the ‘MergeSeurat’ function. The gene expression profiles of each single cell were then merged together yielding 56 082 cells. Then coming to the quality control routine, a cut-off value of 300 and 4000 unique molecular identifiers (UMIs) were used to select single cells for further analysis. Cells with potential mitochondrial content >10% were removed. Next cells outside the 5th and 95th percentile of the distribution of the number of detected genes were discarded. Then we filtered out doublets, which were identified using DoubletFinder [20]. Finally, we obtained 45 229 cells. The data were normalized for library size by a scale factor of 10 000 and log-transformed using the ‘NormalizeData’ function in Seurat, followed by ‘ScaleData’. Highly variable features were determined using the function ‘FindVariableFeatures’ from Seurat. Principal component analysis (PCA) was first performed to obtain a small number of principal components as input to the uniform manifold approximation and projection (UMAP) algorithm. The number of principal components was then chosen at the ‘elbow’ of the plot where a substantial drop was observed in the proportion of variance explained.

### 2.3. Batch correction and UMAP plot generation

In order to remove any potential batch effects between TL and FL samples, we ran the canonical correlation analysis (CCA) [19] that enhances the clustering and the UMAP visualization more biologically meaningful and less driven by batch-specific variations. First, we split the dataset into two groups according to the brain region before eliminating the batch effect. Functions used for this step were ‘FindIntegrationAnchors’ and ‘IntegrateData’ from Seurat. Then, we ran ‘ScaleData’, ‘RunPCA’, ‘FindNeighbors’, ‘RunUMAP’ and ‘FindClusters’. To determine the optimal cluster resolution, we first clustered the data on a range of resolutions (from 0.1 to 2, with steps of 0.2). Then, we plotted the number of obtained clusters versus the resolution and chose a resolution (i.e. 0.3) for which the number of clusters remained constant across multiple resolutions. For visualization, we ran ‘DimPlot’ in Seurat to generate UMAP plot. To test the robustness of batch correction, we also set the Seurat object without batch removal by using the functions: ‘ScaleData’, ‘RunPCA’, ‘FindNeighbors’, ‘RunUMAP’ and ‘FindClusters’.

### 2.4. Cell type identification and KEGG pathways annotation

Differential gene expression analyses between cell clusters were performed using logistic regressions on the integrated normalized counts, by the ‘FindAllMarkers’ function in Seurat with the following settings: assay = ‘RNA’, only.pos = TRUE, test.use = ‘LR’, min.pct = 0.1, logfc.threshold = 0.25. This procedure was used for subcluster classification. Each cluster was then aggregated into main cell type groups by comparing the top 20 genes in each cell type with the known markers [21]. To corroborate the cell classification, we also used the BRETIGEA package, which provides a well-validated set of brain cell type-specific marker genes derived from multiple types of experiments, including marker specificity, enrichment and absolute expression [22]. A hypergeometric test was used to test the overlap between our data-driven markers and the top cell type markers from BRETIGEA. To identify the subcluster-specific genes within EX and INH, differential gene expression analysis between subclusters was performed. We assigned EX subclusters to respective cortical layers by evaluating the expression of layer-specific markers identified by Lake *et al*. [23]. INH formed two major types (branches), associated with different developmental origins in caudal ganglionic eminence (CGE) and middle ganglionic eminence (MGE), respectively [24]. The MGE included PVALB, SST subclasses and the CGE contained LAMP5, VIP subclasses [25]. To interpret the differential gene expression results we carried out KEGG pathway enrichment. To annotate the specific functions of each cell type or neuron-subcluster, we ranked all the differentially expressed genes (DEG) between clusters detected above by avg_log_2_FC in descending order and then input them into ‘gseKEGG’ function of the clusterProfiler [26] package in R. To remove the redundancy in these pathway annotations, the significant pathways (BH-adjusted *p* < 0.05) were then merged and clustered using the default clustering method in pheatmap package.

### 2.5. Differential expression analysis in FL versus TL and pathway enrichment analysis

**Figure 1.**
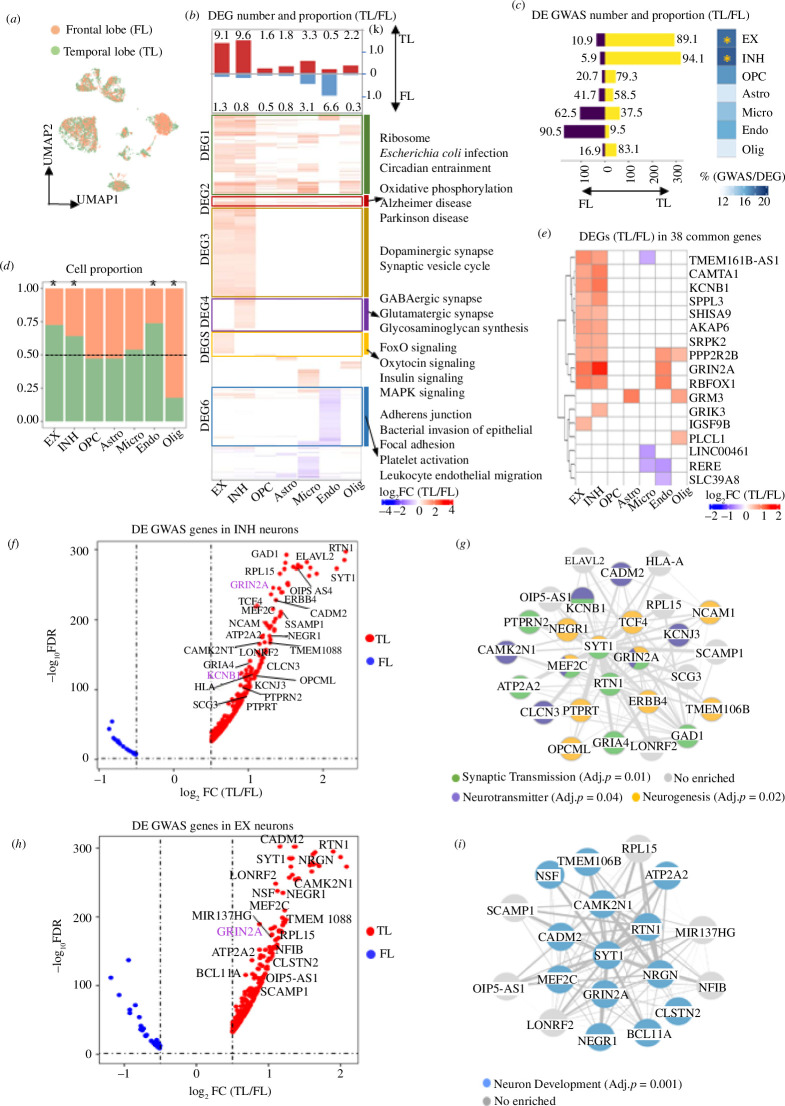
GWAS-associated genes for neuropsychiatric disorders are enriched for neuronal expression in the TL. (*a*) UMAP plot showing how the two regions overlap after batch correction (see §2). (*b*) Bar plot showing the cell type proportions in TL and FL samples. Asterisks mark significantly different proportions by chi-squared test (*p* < 0.05). (*c*) Number of DEGs (absolute log_2_FC > 0.5 and FDR < 0.05) between FL and TL, split by upregulated in TL and FL, respectively. For each cell type, the proportion (%) of DEGs to the total number of expressed genes is indicated (top). Hierarchical clustering of log-FC of DEGs between FL and TL identified in each cell type, which are grouped into six clusters according to the KEGG pathways annotation (BH-adjusted *p* < 0.05) (bottom). (*d*) Number of GWAS genes that are DE between FL and TL, split into upregulated in TL (yellow) and in FL (purple), respectively. Numbers represent the ratio (%) of the upregulated DE GWAS genes in each region over the total DE GWAS genes (left). Proportion of all DE GWAS genes over the total number of DEGs in each cell type, and the significance of enrichment in each cell type by hypergeometric test (right). *BH-adjusted *p* < 0.05. (*e*) Heatmap showing the log_2_FC (TL/FL) of DEGs (absolute log_2_FC > 0.5 and FDR < 0.05) between FL and TL among 38 GWAS genes (detected in seven neuropsychiatric diseases). (*f*,*h*) Volcano plots displaying GWAS gene expression in EX (*h*) and INH (*f*) neurons, respectively. Genes with log_2_FC > 1 and GWAS *p* < 10−8 are highlighted. Genes with colour highlight belong to the 38 common genes detected in seven disorders. (*g*,*i*) Gene networks describing the interaction of genes which are highlighted in the corresponding volcano plots. Edges represent gene–gene co-expression in the specific cell type. Each node (gene) in the network is coloured according to the significant GO functional enrichment (BH-adjusted *p* < 0.05).

For each group of cells, we extracted counts from the processed snRNA-seq data. In order to remove the bias from the imbalance proportion of the two regions, we did downsampling for each cell type to make the cell number consistent across the brain regions. Then, we performed differential expression using edgeR (v. 3.20.8) to identify TF/FL region-associated genes (threshold of absolute log-fold change (FC) > 0.5 and false discovery rate (FDR) < 0.05) at the level of individual genes across the individual cells. EdgeR which has been extensively benchmarked alongside other widely used statistical methods and showed higher performance [27,28]. EdgeR uses an empirical Bayes framework to estimate dispersion and accounts for overdispersion, which helps to stabilize the variance estimates even when sample sizes are small. We performed differential expression (DE) analysis for each cell type or subtype by inputting the count after doing CCA. In the analysis, ‘calcNormFactors’ was used to adjust for varying sequencing depths (due to differing library sizes) as well as minimize the log-FC between the samples for most genes. We the used ‘estimateDisp’ function to estimate common dispersion and tagwise dispersions, and, lastly, the grouped data (FL versus TL) were input to ‘glmQLFTest’ function. After obtaining all the DEGs in each cell type, we combined them together as a matrix and performed hierarchical clustering of the FC (TL/FL) and further annotated the genes function by functional enrichment analysis by ‘enrichKEGG’ in clusterProfiler (threshold for significance: adjusted *p* < 0.05) [26]. Combining the results of the hierarchical clustering with the functional enrichment, we identified six clusters of DEGs that show (1) cell type-specific gene expression patterns between FL and TL and (2) a coherent biological function (reported in figure 1*c*). The thresholds used for finding the DEGs and the enriched pathways in each neuron-subcluster are same as the above analysis. To test the robustness of batch correction, we repeated the same DE analysis by edgeR using the Seurat object without doing CCA. We used Fisher’s test to check the significance of the overlapped DEGs between with and without batch correction.

### 2.6. Enrichment of neuropsychiatric disease GWAS genes in brain cell clusters

**Figure 2.**
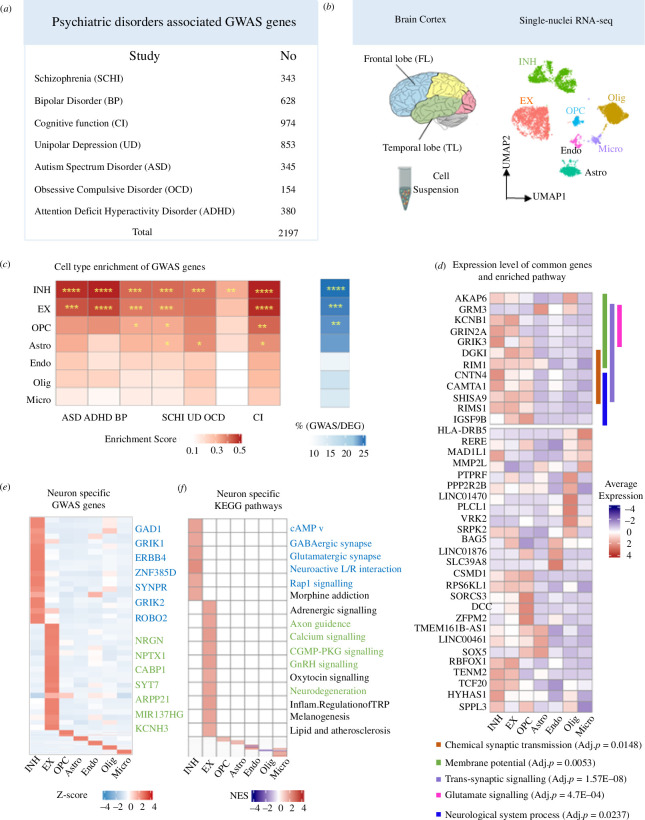
GWAS-associated genes for neuropsychiatric disorders are enriched for neuronal expression. (*a*) Number of GWAS genes for neuropsychiatric disorders extracted from NHGRI-EBI GWAS catalogue. (*b*) Schematic diagram displaying the TL and FL brain neocortex (left). UMAP plot showing all main cell types identified in the snRNA-seq analysis: astrocyte (Astro) oligodendrocyte (Olig), microglia cells (Micro), endothelial cells (Endo), oligodendrocyte progenitor cells (OPC), EX and INH (right). More details are found in electronic supplementary material, figure S1. (*c*) Cell type enrichment score for expression of neuropsychiatric-associated GWAS genes grouped by disease (left). Proportion of GWAS genes within marker genes of each type with significance (BH-adjusted *p*-value) of the hypergeometric test labelled by asterisks (right): Significance (*p*-value) thresholds: *0.01−0.05, **0.001−0.01, ***0.0001−0.001, ****<0.0001. (*d*) Heatmap showing the specific marker genes that are also GWAS-associated genes in EX (green) and INH (blue), respectively. (*e*) Gene set enrichment analysis (GSEA) of cell type-specific differential expression in EX and INH (BH-adjusted *p* < 0.05). Pathways in green and in blue are contributed by GWAS genes in EX and INH, respectively. (*f*) Average (normalized) expression level of 38 genes (detected in seven neuropsychiatric diseases) in each cell type; the significant (non-redundant) Gene Ontology (GO) biological processes (BH-adjusted *p* < 0.05) for these 38 genes are also indicated by different colours (vertical bars) and annotated at the bottom.

To identify whether the neuropsychiatric disease genes identified by GWAS show cell type-specific association or regional alterations between FL and TL, we downloaded a list of GWAS candidate genes related to bipolar disorder (BP, EFO ID: MONDO_0004985), autism spectrum disorder (ASD, EFO ID: EFO_0003756), schizophrenia (SCHI, EFO ID: MONDO_0005090), attention deficit hyperactivity disorder (ADHD, EFO ID: EFO_0003888), obsessive-compulsive disorder (OCD, EFO ID: EFO_0004242), unipolar depression (UD, EFO ID: EFO_0003761) and cognitive function (CI, EFO ID: EFO_0008354) from the NHGRI-EBI GWAS catalogue [29]. We considered the GWAS loci in non-intergenic regions, which have been reported in published studies and that pass the NHGRI-EBI GWAS catalogue eligibility criteria. We first selected single nucleotide polymorphisms (SNPs) with gene associations *p*‐value ≤ 10^−5^. After mapping SNPs to genes using the SNP–gene annotation from the NHGRI-EBI GWAS DB, when multiple SNPs are found in the same gene we focus on the SNP with the lowest *p*‐value. Finally, we selected GWAS genes expressed in all cells that are inside the 10th and 90th percentile of the distribution of the number of genes detected and the number of UMIs in our single-cell RNA-seq data. A summary of the number of GWAS genes selected in each disease is shown in figure 2*a* flowchart of the GWAS genes selection in electronic supplementary material, figure S1*a*.

To calculate the enrichment of genetic risk associated with psychiatric disorders, we used a hypergeometric test for the overlap between cell type-specific genes (DEGs between one cell with other cell types, log_2_FC > 0.5, adjusted, *p* < 0.05) and the variant-mapped genes for each disease, which is widely used to evaluate the enrichment of genetic risk genes [30,31]. First, the SNPs and the SNP-mapped genes retrieved from NHGRI-EBI GWAS DB are filtered by the step in electronic supplementary material, figure S1a. For the SNPs mapping to multiple genes, we use all the mapped genes as input. The background gene set used in the hypergeometric test was genes expressed in each cell type (or subtype) that are inside the 10th and 90th percentile of the distribution of the number of genes detected in our single-cell RNA-seq data; the *p*-values of enrichment were corrected for multiple testing using the Benjamini–Hochberg (BH) procedure.

### 2.7. Functional network of GWAS genes for neuropsychiatric diseases

After filtering the GWAS genes, we explored the enriched biological pathways. We carried out KEGG pathway enrichment analysis by ‘enrichKEGG’ function of the clusterProfiler [26] package in R. The significant pathways (BH-adjusted *p* < 0.05) were then grouped into multiple pathway clusters according to their high-level pathway name entry in the KEGG database. Many genes are shared by different pathways and this property was assessed by means of the Jaccard similarity coefficient. For each pathway pair, the Jaccard similarity coefficient was calculated by assessing the number of overlapping genes between two pathways divided by the number of union genes in two pathways. The pathways’ relationships are represented as a network, where each edge connecting any two pathways is proportional to the Jaccard similarity coefficient; the resulting network was plotted by Cytoscape software (v. 3.9.0).

### 2.8. Comparison with genes dysregulated in diseased brain tissue

**Figure 3.**
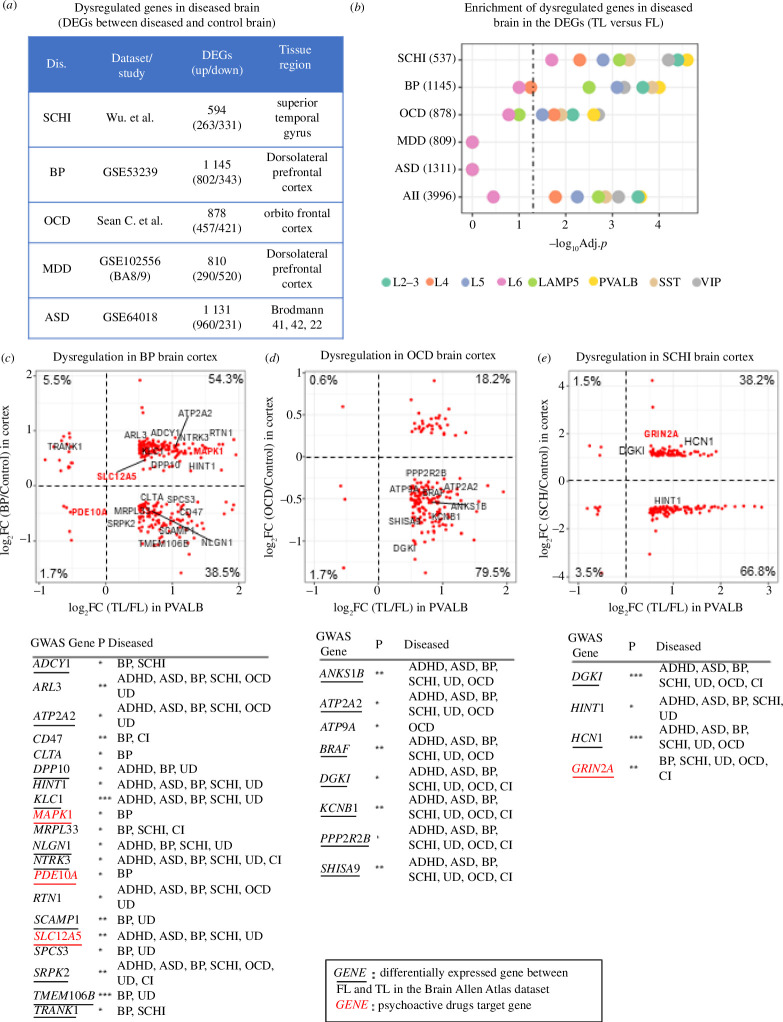
Genes expressed in PVALB neurons from the TL are also dysregulated in neuropsychiatric diseased brain tissue. (*a*) Details about the DEGs (absolute log_2_FC > 0.25, FDR < 0.05) identified in the brain of neuropsychiatric disease patients (see §2 for details). (*b*) Bubble plot showing the enrichment of DEGs (absolute log_2_FC > 0.5, FDR < 0.05) between TL and FL (for each neuron subtypes) in the set of DEGs detected in the brain of neuropsychiatric disease patients. Numbers in brackets are number of DEGs (diseased brain versus control brain). Dotted vertical line indicates the threshold for statistical significance of enrichment (BH-adjusted *p* = 0.05). (*c–e*) Scatter dot plots showing the relationship between log_2_FC (TL/FL) of PVALB-expressed genes (*x*-axis) and log_2_FC (diseased/control brain) (*y*-axis) for the set of genes significantly overlapping genes. Three scatter plots for BP (*c*), OCD (*d*) and SCHI (*e*). For each disease, the genes highlighted in each panel are GWAS-associated genes with GWAS *p* ≤ 10^−5^, and the numbers in each quadrant are the proportion of genes over the total number of DEGs in T/FL and in diseased/control brain. Each Scatter dot plots is accompanied by a table showing the associated neuropsychiatric disease for each gene by GWAS: SCHI, BP, CI, UD, ASD, OCD and ADHD. Bold red font, GWAS-associated genes that are also psychoactive drugs targets; underlined genes are DEGs between FL and TL in the Allen Brain Atlas dataset (BH-adjusted *p* < 0.05). GWAS-association *p*-values: *10^−8^–10^−5^; **10^−10^–10^−8^; ***<10^−10^.

To identify genes dysregulated in the brain tissue from patients with neuropsychiatric disease, we retrieved public datasets from the Gene Expression Omnibus (GEO) database, as well as retrieving preprocessed lists of DEGs from original publications. For the GEO data, we downloaded three bulk RNA datasets related to: major depressive disorder (MDD) (GSE102556, BA 8/9) [32], ASD (GSE64018) [33], BP (GSE 53239) [34], respectively. For OCD and SCHI, we used the list of DEGs from Refs. [35] and [36] respectively, because (i) the authors found no significant differences in gene expression due to other factors and covariates tested [36] and (ii) the potential effect of covariates was accounted for using generalized linear regression models in [35]. Each of the dataset comes from a part of FL or TL of the brain cortex, as detailed in figure 3*a*. For GSE102556 and GSE64018, we downloaded the normalized FPKM matrix from the GEO website as input to obtain the DEG. For GSE53239, since only count data are provided on the website, we standardized it with the FPKM method to ensure consistency. We could not include any covariates when re-analysing these RNA-seq data as these were not provided in GEO. ‘edgeR’ was used to perform differential expression analysis by comparing gene expression in control group versus disease group. After correction of multiple testing, genes with BH-adjusted *p*‐value < 0.05 and absolute log_2_FC > 0.25 were selected as disease-associated genes. For each disorder, the list of DEGs (disease-associated genes) was tested for overlap with the set of DEGs between FL and TL identified here. The significance of the overlap was assessed by hypergeometric test (background: all genes expressed in each cell type or subtype) and the *p*-values were corrected using the BH approach, as indicated.

### 2.9. Gene co-expression networks of GWAS genes enriched for expression in neurons

**Figure 4.**
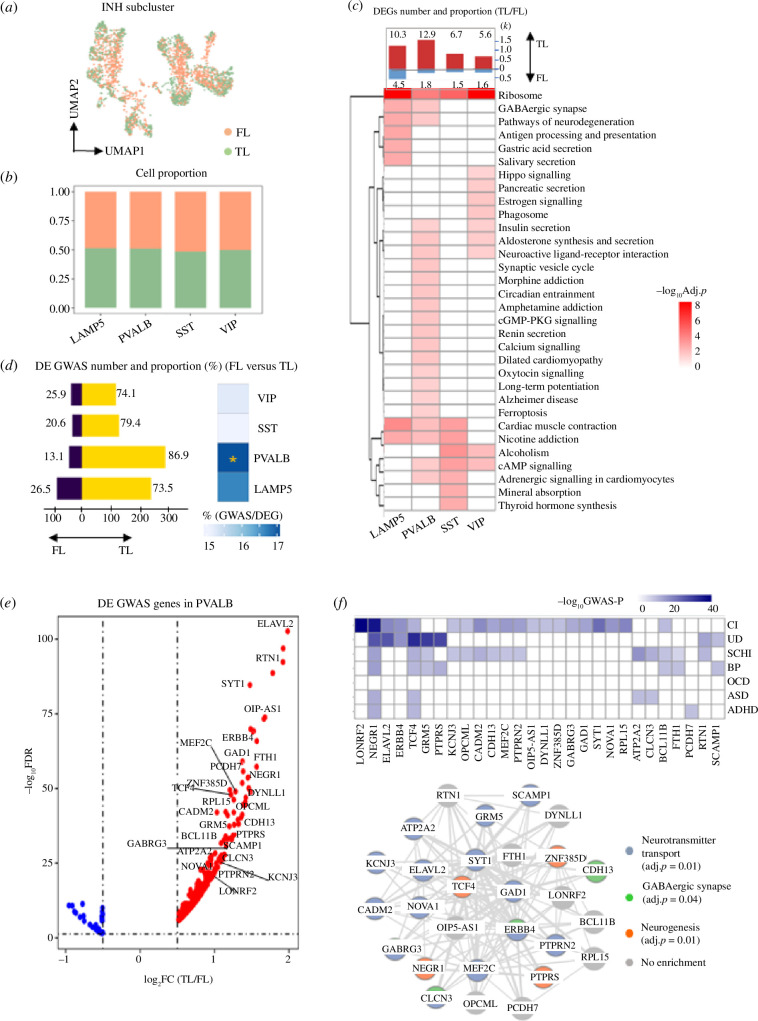
GWAS-associated genes for neuropsychiatric disorders are specifically enriched for PVALB expression in the TL. (*a*) UMAP plot showing the FL and TL distribution to INH neurons. (*b*) Cell proportion of FL and TL in each subcluster. (*c*) Number of DEGs between FL and TL (absolute log_2_FC > 0.5 and FDR < 0.05) split by genes upregulated in FL and TL, respectively. The proportion (%) of DEGs over the total number of expressed genes is indicated for each subcluster (top). Heatmap exhibit the KEGG pathways enriched by DEGs between FL and TL (BH-adjusted *p* < 0.05). (*d*) Number of GWAS genes that are DE between FL and TL, split by upregulated in TL (yellow) and FL (purple), respectively. Numbers represent the proportion (%) of the upregulated DE GWAS genes in each region over the total number DE GWAS genes (left). Proportion (%) of all DE GWAS genes over the total of DEGs in each cell type; significant enrichment (by hypergeometric test) is indicated (right). *BH-adjusted *p* < 0.05. (*e*) Volcano plot showing log_2_FC (TL/FL) for the GWAS-associated genes in PVALB cluster. Subset of 28 GWAS genes with log_2_FC > 1 and GWAS *p* < 10^−8^ are highlighted in the graph. (*f*) Heatmap showing the GWAS *p*-values for the 28 genes in each disease (top). Gene network describing the interaction of the 28 GWAS genes. Edges represent gene–gene co-expression in PVALB neurons. Each node (gene) in the network is coloured according to the significant GO functional enrichment (BH-adjusted *p* < 0.05).

For selected subset of GWAS genes enriched in neuronal subtypes we derive cell type-specific gene co-expression networks (figures 1*g*,*i* and 4). We used Spearman correlation to calculate the connection between each gene, where the correlations in gene expression were calculated across cells in the same compartments (e.g. within inhibitory neurons). We used the Spearman correlation as this performs better than the traditional Pearson correlation and has higher accuracy and reproducibility for network reconstruction using single-cell data [37]. All the gene–gene connections with *p* < 0.05 for the Spearman correlation were deemed significant and retained to represent the gene network, which was plotted by Cytoscape software (v. 3.9.0). In the network graph, each edge connecting any two genes represents the Spearman correlation.

### 2.10. Enrichment of psychoactive drug-target genes in brain cell types

To evaluate the response to psychotropic medications in each brain cell type, drug-target gene sets were originally retrieved from DSigDB (http://dsigdb.tanlab.org/DSigDBv1.0/). Seven drug classes, which have been approved by clinical trials and are widely used in the clinic with good efficacy in the treatment of psychiatric disorders, have been considered here: selective serotonin reuptake inhibitor (SSRI), dopamine receptor agonists (DA), tricyclic antidepressant (TCA), benzodiazepines (BZD), monoamine oxidase inhibitor (MAOI), GABA receptor agonists (GABA) and adrenergic receptor agonist (ARA). With respect to depression and bipolar disorder, we included classical antidepressants (SSRI, TCA, MAOI and BZDs), and they also contribute to schizophrenia treatment. ARA is used in bipolar, autism and depression treatment. With respect to autism and ADHD, OCD, GRA and GABA are commonly used. Although DA is mainly used for Parkinson patients, many studies indicate its efficiency in BP and schizophrenia too [38,39]. The enrichment of drug-target genes was performed in the same way used to assess the enrichment of GWAS genes (detailed above). All gene targets for each psychoactive drug class are listed in electronic supplementary material, table S4.

### 2.11. Replication analyses in human Allen Brain Atlas

The human multiple cortical areas SMART-seq data [21] were downloaded from https://portal.brain-map.org/atlases-and-data/rnaseq/human-multiple-cortical-areas-smart-seq, which contains single-nucleus transcriptomes (from 49 495 nuclei) from four donors, each with six cortical regions profiled (middle temporal gyrus (MTG); anterior cingulate cortex (CgG), known as the medial PFC; primary visual cortex (V1C); primary motor cortex (M1C); primary somatosensory cortex (S1C); and primary auditory cortex (A1C)). The data matrix was input into Seurat to construct the Seurat object for downstream analysis. ‘Findmarkers’ function was used here to calculate the main cell type or neuronal-subtype identical genes (BH-adjusted *p*‐value < 0.05 and absolute log_2_FC > 0.25). We used edgeR package in R to obtain the DEGs between regions, such as MTG versus A1C+CgG+M1C+S1C+V1C, A1C versus MTG+CgG+M1C+S1C+V1C, etc. Genes with BH-adjusted *p*‐value < 0.05 and absolute log_2_FC > 0.5 were selected as regionally differential genes. The significance of GWAS genes enrichment within different cortical regions was determined by hypergeometric test (BH-adjusted *p*‐value < 0.05), where regionally differential expressed genes are deemed representative of each region. The background gene set was all genes expressed in each cell type or subtype. We also performed the hypergeometric test to calculate the significance of overlapped DEGs between our dataset and the DEGs obtained derived from Allen Brain Atlas data (DE analysis by edgeR, as indicated before). To calculate the percentage of dysregulated genes from bulk RNA-seq data in the Allen Brain Atlas data (reported in electronic supplementary material, figure S11*b*–*f*), for each of the six cortex regions we considered a gene to be expressed if number of cells expressing a gene is inside the 10th and 90th percentiles of the distribution of the number of cells expressing all genes.

### 2.12. RNA isolation and quantitative real-time PCR

Total RNA from human brain tissue was extracted using the RNAeasy Animal RNA Isolation Kit with Spin Column (R0027; Beyotime Biotechnology). In brief, the tissue was added to pre-cooled lysis buffer in an ice bath, homogenized with an electric homogenizer and the homogenate was left at room temperature for 3–5 min. After adding binding solution and vortexing, the mixture was centrifuged at 12 000 ×*g* for 30 s. After discarding the liquid, washing steps were performed. The purified total RNA was obtained by centrifuging at 16 000 ×*g* for 30 s after elution. Subsequently, 400 ng total RNA from each sample was reverse-transcribed into cDNA using HiScript III RT SuperMix for qPCR (+gDNA wiper) (R323-01; Vazyme, Nanjing, China). Real-time quantitative PCR analysis was conducted using ChamQ Universal SYBR qPCR Master Mix (Q711-02; Vazyme) on the QuantStudio 7 system (Thermo Fisher) and 7500 system (Applied Biosystems). The 2^−∆∆CT^ method was employed to calculate relative transcript abundance, with GAPDH as the normalization reference. Results are presented as mean ± s.d. from three replicates. The following primers were used in this study: GRIN2A, forward: 5′-CGGCAGAACTCCACGCACTG-3′, reverse: 5′-GGCAGGCATCGCACTTGAAGG-3′; SLC12A5, forward: 5′-CAACAGCACCGACACAGAGAAGG-3′, reverse: 5′-ACTGAGCAAGGAGGACACCATAGG-3′; and GAPDH, forward: 5′-GTGGACCTGACCTGCCGTCTAG-3′, reverse: 5′-GAGTGGGTGTCGCTGTTGAAGTC-3′. The significance of mRNA level differences was calculated by *t*‐test (two-tailed). Sample information is included in electronic supplementary material, table S3.

## 3. Results

### 3.1. Susceptibility genes for neuropsychiatric disorders share common pathways

To investigate which functional and cellular processes are dysregulated in neuropsychiatric disorders, we collected GWAS data from seven psychiatric disorders (BP, ASD, SCHI, ADHD, OCD, UD and CI) (figure 2*a* and electronic supplementary material, table S1). To confirm the association of these diseases with dysregulated processes in TL and/or FL, we conducted a separate enrichment analysis of GWAS genes across six cortical regions using the SMART-Seq data from the Allen Brain Atlas. GWAS genes were significantly enriched in both TL and FL (adjusted *p* = 3.6 × 10^−5^ and adjusted *p* = 0.04, respectively) (electronic supplementary material, figure S1*e*), supporting the connection between these diseases and dysregulated processes in these brain regions. A subset of 38 genes was detected by GWAS in all seven neuropsychiatric disorders (electronic supplementary material, figure S1*b*), and these were enriched for KEGG pathways and biological processes, including synapse related as well as neuron degeneration diseases, endocrine function and neuronal signalling pathways (electronic supplementary material, figure S1*c*). We also annotated a set of ‘core pathways’ shared between at least four neuropsychiatric diseases (electronic supplementary material, figure S1*d* and table S2). Among the most common pathways, synaptic dysfunction and neurodevelopmental conditions (*blue circle* in electronic supplementary material, figure S1*d*) have been reported to be altered in the postmortem brain in ASD [40]. Besides, exome sequencing studies suggested the role of post-synaptic density in SCHI [41,42]. Many signalling transduction-associated pathways, including AKT, AMP and AMPK, play a role in higher mental functions such as mood, neurogenesis and neurotransmitter [43,44] (*green circle* in electronic supplementary material, figure S1*d*). We also found that GnRH-related pathways belonging to endocrine system being enriched in most of the disorders (*pink circle* in electronic supplementary material, figure S1*d*); hormones regulate ovary and testicular function in mammals and changes in gonadotropin levels are known to impact mood and contribute to the development of affective disorders [45]. These analyses show that GWAS genes for heterogeneous neuropsychiatric diseases impact commonly shared pathways and broad brain functions important for disease pathobiology. To shed light on potential regional and cell type-specific functions we carried out single-cell profiling in the brain.

### 3.2. Susceptibility genes for neuropsychiatric disorders are enriched for expression in neuronal cell types

We sequenced single nuclei from frozen brain cortex tissues of three subjects (one sample from the dorsolateral PFC in FL and two samples from temporopolar lobes) with no history of neuropsychiatric disorder (figure 2*b* and electronic supplementary material, table S3), and, after filtering out poorly sequenced nuclei and potential doublets, we identified a total of 45 229 cells (electronic supplementary material, figure S2*a*). UMAP identified seven main cell clusters, which were also manually corroborated using well-known marker genes (electronic supplementary material, figure S2*b*). Excitatory neurons were the most abundant (38.3%) cell type, followed by inhibitory neurons and oligodendrocyte (23.6% and 22.1%, respectively), which is consistent with previously reported cell proportions in cortical tissue areas [46] (electronic supplementary material, figure S2*c*,*d*). Excitatory neurons were characterized by the expression of ENC1 and SLC17A6 [47], while inhibitory neurons were identified by GABAergic (GAD2 and GRIK1) and dopaminergic neurons by TH [47–49]. Immune cells displayed a specific expression of well-known markers, e.g. CD74 in microglia [50], RGS5 in endothelial cells [51]. PTPRZ1 and BCAN were highly expressed in OPC [52,53], while oligodendrocytes are characterized by MOBP and MBP [54] expression. Expression of AQP4 was characteristic for astrocytes [55]. Our cell cluster classification was confirmed by the overlap with known cell markers derived from a previous study [30] (electronic supplementary material, figure S2*f*). Pathway enrichment analysis confirmed functional specialization of different cell types (electronic supplementary material, figure S2*e*), supporting the fidelity of our brain cell types clustering.

Next, we integrated the neuropsychiatric disorders GWAS data with brain snRNA-seq data to annotate the cell type-specific expression and cellular mechanism of the disease susceptibility genes, and detail whether these genes exhibit expression differences between FL and TL. Overall, all genes associated with disease by GWAS (thereafter named ‘GWAS genes’) (*n* = 2197, combined across seven diseases) showed the highest enrichment for expression in EX, INH and OPC (adjusted *p* = 8 × 10^−9^, 10^−4^, 3 × 10^−3^, respectively, hypergeometric test, figure 2*c*). GWAS genes accounted for 21.7% and 24.6% of the neuronal marker genes in EX and INH, respectively. Considering each disease separately, we found significant enrichment for all diseases in INH markers and for five diseases in EX markers (figure 2*c*). We also identify GWAS genes such as *NRGN*, *CABP1* and *NPTX1* as cell type markers for EX, and *GRIK1*, *GAD1* and *ERBB4* for INH (figure 2*d*). On the whole, these results suggest neuronal cell type might play a key role in mediating the genetic susceptibility to several neuropsychiatric disorders [56]. To validate our findings, we performed the enrichment analysis of GWAS genes in cortical cell types using the SMART RNA-seq from Allen Brain Atlas [21]. Consistent with the results in our data, ADHD, BP, CI, UD associated GWAS genes were strongly enriched in INH, while ADHD, BP were enriched in EX neurons (electronic supplementary material, figure S8*a*).

The pathways impacted by the GWAS genes expressed specifically in EX and INH neurons are consistent with the four common pathway groups enriched in the whole set of GWAS genes (‘core pathways’ in electronic supplementary material, figure S1*b*,*d*), which is in keeping with previous reports [57]. The main pathways are axon guidance, calcium signalling process, GnRH signalling pathway and neurodegeneration diseases for the EX-expressed GWAS genes, and GABAergic synapse, neuroactive L/R interaction and depression for the INH-expressed GWAS genes (figure 2*e*). When we looked at the set of 38 GWAS genes shared across all seven psychiatric disorders, not surprisingly, we found a significant enrichment for expression only in EX and INH neurons (adjusted *p*-value of 0.004 and 6.4 × 10^−5^, respectively, hypergeometric test). Furthermore, a subset of 12 genes (most highly expressed in EX and INH neurons) shows specific and non-redundant enrichment for GO biological processes. Specifically, *GRIN2A*, *KCNB1*, GRM3 and *GRIK3* are enriched in synaptic signalling and glutamate signalling; *DGK1* and *RIM1* are related to synaptic transmission, *SHISA9* and *RIMS1* contribute to neural processes, while *CNTN4* and *CAMTA1* are involved in both (electronic supplementary material, figure S1*c*).

### 3.3. Extensive cell type-specific transcriptional changes in brain cortex between FL and TL

We then investigated the cell types and expression changes between FL and TL (figure 1*a*). EX, INH and Endo showed the highest cell proportion in TL, while Olig were overrepresented in FL (figure 1*b*). We found the highest number of DEGs between FL and TL in EX and INH, with most genes being upregulated in TL (figure 1*c*, top panel). The larger number of DEG in TL versus FL in INH and EX was not affected by the higher proportion of INH and EX neurons in TL (see §2). We also identified six clusters of DEGs (electronic supplementary material, figure S3) that show (i) coherent expression changes between FL and TL and (ii) enriched for the same biological functions or pathways (figure 1*c*, bottom panel). These patterns reflect coherent changes in the expression of genes, which were mostly upregulated in TL, especially in EX and INH. In addition, we show that the DEGs (TL/FL) in EX, INH, astrocyte, oligodendrocyte, OPC, microglia from our data show a significant overlap with the DEGs (TL/FL) obtained by the analysis of the Allen Brain Atlas dataset (electronic supplementary material, figure S9*d*), supporting the reliability of our results.

Resolving these regional gene expression changes at the single-cell level might help elucidating the functional context of GWAS genes for neuropsychiatric disorders. We therefore investigated whether the GWAS genes overlap with the DEGs between FL and TL identified in each cell type. The DEGs in EX and INH showed the largest overlap with GWAS genes (adjusted *p*‐value < 0.05, hypergeometric test) and most of them are upregulated in TL compared to FL (figure 1*d*, bar plot). From the results of the Allen Brain Atlas data, INH in TL is highly associated with BP, SCHI, UD and EX in TL is associated with ADHD, CI and UD (electronic supplementary material, figure S8*b*).

Of the 38 common GWAS genes, 17 (45%) showed significant changes between TL and FL, with most genes (>89%) being upregulated in TL in EX and INH, including *KCNB1*, *GRIN2A* and *CAMTA1* (figure 1*e*), and enriched for biological processes such as regulation of neurological system, glutamate signalling and synaptic signalling (electronic supplementary material, figure S4*h*).

We then explored the relationship between the significance of GWAS associations (GWAS *p*‐value) and the degree of change (FC) in gene expression between TL and FL in EX and INH. While we found no obvious and consistent correlations between GWAS gene significance and FCs (electronic supplementary material, figure S4*a–g*), we identified *GRIN2A* and *KCNB1* with highly significant GWAS associations (*p* < 10^−8^) in all seven diseases, and more strongly upregulated TL (FDR < 0.05, log_2_FC (TL/FL) > 1) in either both INH/EX (*GRIN2A*) or in INH (*KCNB1*) (figure 1*f,h*). The cell type-specific gene co-expression networks describing the interactions between the most significant GWAS genes (*p* < 10^−8^) were enriched for neurogenesis and neurotransmitter in INH (figure 1*g*), and neuron development in EX (figure 1*i*). Therefore, we showed a significantly high proportion of genetic risk factors for neuropsychiatric disorders are upregulated in neuronal cells from the TL.

### 3.4. Susceptibility genes for neuropsychiatric disorders are mostly enriched in PVALB interneurons from the TL

**Figure 5.**
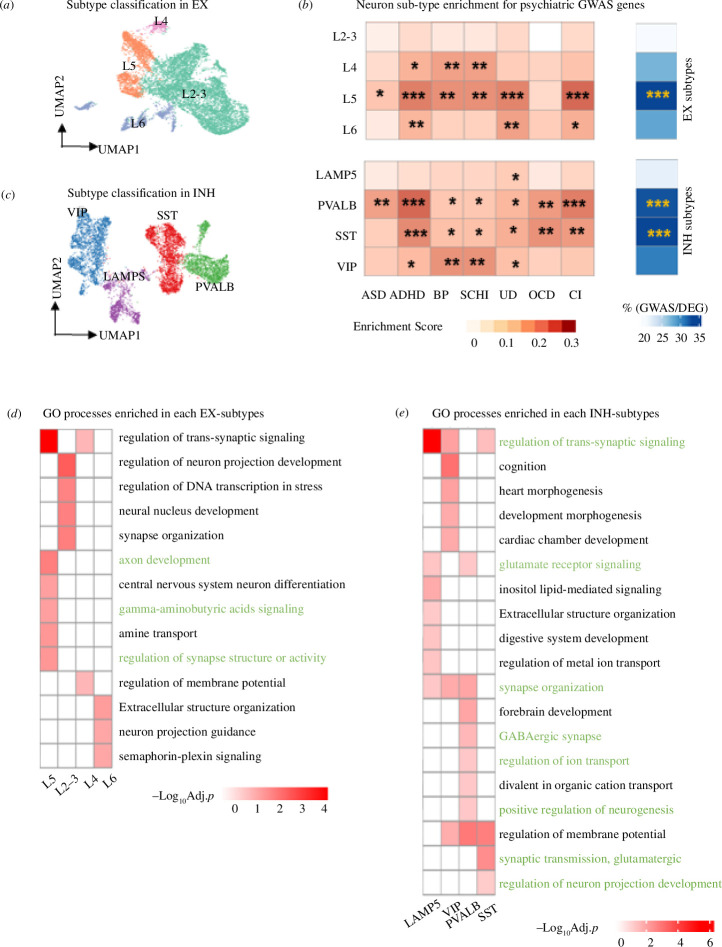
Specific neuronal subtypes show enrichment for GWAS-associated genes for neuropsychiatric disorders. (*a*,*b*) UMAP plot showing the subtypes of EX (*a*) and INH (*b*) neurons. EX neurons are classified into L2–3, L4, L5 and L6. INH neurons are classified into LAMP5, PVALB, SST and VIP. (*c*). Cell type enrichment score for expression of neuropsychiatric disorder associated GWAS genes. Proportion (%) of GWAS genes within marker genes of each cell subtype alongside significance of enrichment (adjusted *p*-value) by hypergeometric test (right). Significance (*p*-value) thresholds: *0.01–0.05, **0.001–0.01, ***0.0001–0.001, ****<0.0001. (*d*,*e*). Enriched GO biological processes (BH-adjusted *p* < 0.05) in each subtype of EX (*d*) and INH (*e*) neurons. Pathways highlighted in green match the pathways identified in GWAS genes (reported in electronic supplementary materials, figure S1 and table S2).

Given the results above we focussed on neuronal cells. Using our single-cell transcriptomic data to identify neuronal cell subtypes, we uncovered EX subtypes L2–3, L4, L5, L6 and INH subtypes SST, PVALB, LAMP5 and VIP (figure 5*a,b*). Neuron subtypes were identified by well-established markers that also enriched for specific functional categories [24] (electronic supplementary material, figure S5*a*,*b*). Supporting the cellular identity of our marker genes, we found that neuronsubtype markers in our data significantly overlap with neuron-subtype markers in the Allen Brain Atlas dataset (electronic supplementary material, figure S5*c,d*). To identify the most relevant neuronal subcluster for a particular disorder, we calculated the percentage of the GWAS genes overlapping with the cell type subcluster-specific markers (figure 5*c*). Overall, we found stronger enrichment in INH than in EX subclusters, with INH-PVALB, SST and EX L5 being the neuronal subclusters mostly enriched for expression of GWAS genes, which accounted for about 33% of the marker genes in each subcell type (figure 5*c*, right panels). The overlap between GWAS genes and neuronal subcluster marker genes is also found at the pathway level. We found biological processes and pathways enriched in the marker genes of the neuronal subclusters (figure 5*d*,*e*, green highlight) which are consistent with those identified in the GWAS genes (electronic supplementary material, figure S1*c*). For example, INH-PVALB and SST were both enriched for processes related to neuron system development, synaptic neurotransmitter and glutamatergic signalling, which are also significantly enriched in GWAS genes (electronic supplementary material, figure S1*d*). To corroborate our findings, a separate enrichment analysis using Allen Brain Atlas data confirmed the significant associations of INH-SST and INH-PVALB with ADHD, BP and UD, while EX-L2–3 was associated with BP, OCD and UD (electronic supplementary material, figure S8*c*).

We went on to investigate the contribution of TL and FL differences on neuronal subclusters. For INH, cells from two regions are well mixed and we found no difference in cell number (figure 4*a*,*b*). Differential expression analysis between TL and FL in INH subclusters identified manifold DEGs, with the PVALB and LAMP5 subclusters having the highest number of DEGs (figure 4*c*, bar plot on the top panel). Similarly, we characterized regional expression differences in EX neurons (electronic supplementary material, figure S6*a*,*b*), and detected many DEGs in EX subclusters, e.g. L5 and L4 (electronic supplementary material, figure S6*c*, bar plot on the top panel). For both neuronal cell types, most of the DEGs were upregulated in TL compared with FL. Functional analysis of the marker genes in each INH subcluster shows enrichment for both common (e.g. ‘ribosome’) and subcluster-specific processes (e.g. synaptic vesicle cycle in PVALB) (figure 4*c*, bottom panel). Among other neuronal cells, the most specific functional enrichment was detected for PVALB subcluster, including signalling factors like GABA, calcium, cAMP and PKG that are essential for structural changes in synapses, dendrites and axons (figure 4*c*, bottom panel), which are consistent with previous reports on structural plasticity differences between FL and TL in PVALB neurons [58]. Long-term potentiation and synaptic vesicle which can impact synapse and axon growth also differ in PVALB cluster [59]. These gene expression changes in functional pathways between TL and FL would be consistent with those reported in neuropsychiatric diseases, for example, changes in long-term potentiation can induce depression, bipolar and schizophrenia [60], degradation of cAMP or cGMP can result in depressive-like behaviours [61]. To test the robustness of batch correction (due to the small sample size), we found the majority of DEGs (2TL versus FL) identified with batch effect correction largely overlap with the DEGs (2TL versus FL) obtained without considering batch effect correction for both major cell types and neuronal subtypes (electronic supplementary material, figure S15*a*,*c*). Moreover, the majority of DEGs with batch correction (2TL versus FL) overlap with the DEGs obtained in individual TL versus FL comparisons (electronic supplementary material, figure S15*b*,*d*)

To investigate whether gene expression differences in FL and TL at the neuronal subcluster level can influence neuropsychiatric disorders genes activity, we looked at the GWAS genes that are DE between TL and FL, and tested for enrichment in each neuronal subcluster. While no enrichment was detected in any EX subcluster (electronic supplementary material, figure S6*d*), we found a significant enrichment of GWAS DEGs (*n* = 327, ~17%; enrichment *p* < 0.05) in INH-PVALB subcluster, with most GWAS genes (~87%) being upregulated in TL (figure 4*d,e*). Specifically, SCHI, BP and UD disorders had the highest enrichment for GWAS genes that are upregulated in TL compared with FL (electronic supplementary material, figure S7*a*), including a subset of 11 GWAS genes that are more strongly expressed in PVALB neurons (electronic supplementary material, figure S7*b*). Using the Allen Brain Atlas dataset, we confirmed that the INH-PVALB in the TL is associated with several psychiatric disorders, such as UD, BP and SCHI, and the results also confirm the relevance of INH-SST in the TL to these three diseases (electronic supplementary material, figure S8*d*). The Allen Brain Atlas dataset revealed additional GWAS enrichments in L2–3 neurons from the primary visual cortex.

A set of 28 genes expressed in PVALB subcluster had robust significance of GWAS association (i.e. *p* < 10^−8^) and sizeable difference in gene expression between TL and FL (i.e. log_2_FC > 1) (figure 4*e*). Overall, we found 28 distinct genes (out of 285 GWAS genes, 9.8%) that were upregulated in PVALB from the TL and robustly associated (GWAS *p* < 10^-8^) with psychiatric disease (figure 4*f*, top panel). We annotated the GWAS genes’ functional connectivity in PVALB cells and derived their PVALB-specific co-expression network (figure 4*f*, bottom panel), which is enriched for neurotransmitter, neurogenesis and GABAergic synapse processes. Dysregulation of these processes has been linked to the pathogenesis of mental diseases, e.g. PV-deficient GABA synapses increased asynchronous release of GABA and shaped behavioural responses, which decreased the level of excitation, as observed in schizophrenia and related psychiatric disorders [62]. To test the sensitivity of the enrichment analysis, we selected the GWAS genes with different *p*-value thresholds: 10^−6^, 10^−7^ and 5 × 10^−8^. These results are largely consistent with those obtained using a *p*-value of 10^−5^. With respect to the neuronal subtype, we found stronger enrichment in INH than in EX subclusters, with INH-PVALB, SST and EX L5 being the neuronal subclusters mostly enriched for expression of GWAS genes (electronic supplementary material, figure S14*a–f*).

### 3.5. Psychoactive drugs target genes are enriched in PVALB interneurons from the TL

Additionally, we investigated the transcriptional impact (aka ‘vulnerability’ [18]) of common and approved by clinical trials of psychoactive drugs (SSRI, DA, TCA, BZDs, MAOI, GABA and ARA) on genes expressed in specific cell types and subcell types from the TL and FL [18]. To this aim, we used the drug-target dataset from DsigDB and assessed the degree to which the gene targets of each drug are expressed in specific cells and subcell types (see §2). Drug-target genes for DA BDZs and GABA were strongly enriched for high expression (BH-adjusted *p* < 0.05) in PVALB cells specifically from the TL (electronic supplementary material, figure S10*a,b*). Overall, we report 177 (out of 1215, 17.6%) genes upregulated in PVALB from TL that are responsive to many of the psychoactive drugs considered here (electronic supplementary material, table S4). We also found a significant enrichment for drug-target genes in other neuronal subtypes (VIP from TL) and astrocytes (mostly in TL). However, compared with other neuronal subtypes, the expression level of drug-target genes in TL of PVALB, SST, L5 were also significantly associated with the degree of enrichment (significance of Pearson correlation: *p* = 0.047, *p* = 0.026, *p* = 0.044, for PVALB, SST, L5, respectively) (electronic supplementary material, figure S10*h*,*j*, *n*).

These data show that the set of PVLAB genes that are upregulated in TL are also significantly enriched for both GWAS genes and psychoactive drug targets, highlighting the importance of regional differences (and especially TL) for neuropsychiatric disorders genes and response to psychoactive drugs in PVALB.

### 3.6. Regional gene expression differences in PVALB neurons are associated with gene dysregulation in neuropsychiatric disease

To assess if regional differences in gene expression in healthy brain can be relevant to disease-associated changes in brain tissue, we leveraged publicly available bulk RNA-seq data generated from neocortex tissue of diseased and control patients (figure 3*a*), including BP, SCHI, ASD, OCD and MDD. All the DEGs identified between cases and controls are listed in electronic supplementary material, table S5. We first tested if the DEGs between TL and FL are enriched in the DEGs found in the diseased brain, and found the highest enrichment for neuronal cell types (electronic supplementary material, figure S11*a*). The most significant overlap with the DEGs identified in each brain disease was detected for the PVALB subcluster, particularly for OCD (adjusted *p* = 2.7 × 10^−3^), BP (adjusted *p* = 9.6 × 10^−5^) and SCHI (adjusted *p* = 2.6 × 10^−5^) (figure 3*b*). We also combined all disease-related genes (non-redundant set of DEGs across all diseases, *n* = 4028), which confirmed the strongest enrichment for the DEGs in the PVALB subcluster. Using the Allen Brain Atlas dataset, we showed that disease-dysregulated genes overlapping with DEGs from our data are overall highly expressed in TL and FL compared with other regions from Allen Brain Atlas (electronic supplementary material, figure S11*b–f*). In addition, the DEGs for SCHI, OCD and all diseases combined, showed a significant enrichment in PVALB neurons in the Allen Brain Atlas dataset (electronic supplementary materials, table S6 and figure S12). For the set of overlapping genes, we investigated the relationships between the expression changes in TL/FL (i.e. log_2_FC (TL/FL)) in PVALB and the changes in the diseased brain (i.e. log_2_FC (disease/control)). We observed that most of the PVALB genes that are upregulated in TL are also upregulated in BP (54.3%). In contrast, the majority of genes upregulated in TL showed downregulation in OCD (79.5%) and SCHI (66.8%) brain cortex (figure 3*c–e*). Among the set of DEGs in TL/FL and diseased/control brain tissues, we identified several GWAS genes for neuropsychiatric disorders (tables in figure 3*c–e*), with most of them being in common to at least two disorders. For example, GRIN2A and SLC12A5 are associated with schizophrenia and bipolar disorder, respectively, by GWAS (*p* < 10^−8^) and are targets for psychoactive drugs (DA and ARA); both genes are also consistently upregulated in TL and dysregulated (upregulated) in brain tissue from schizophrenia patients (bold font, tables in figure 3*c–e*), which were confirmed by qPCR (our samples) as well as by independent analysis of Allen Brain Atlas data (electronic supplementary material, figure S12*b*,*c*).

We also compared the regional differences in our dataset with the DEGs in ASD, which were obtained by single-cell RNA-seq data from an existing study [16] comprising 22 ASD and 19 control samples. Except for LAMP5, Endo and L4, ASD-associated dysregulated genes significantly overlap with DEGs between FL and TL in other cell types, especially VIP and astrocytes (electronic supplementary material, figure S16). While PVALB is not the most apparent cluster reflecting regional differences contributing to ASD, we found a moderate association (*R*^2^ = 0.11, *p* = 0.04) between changes in TL/FL and those in ASD/Ctrl (electronic supplementary material, figure S16).

## 4. Discussion

**Figure 6.**
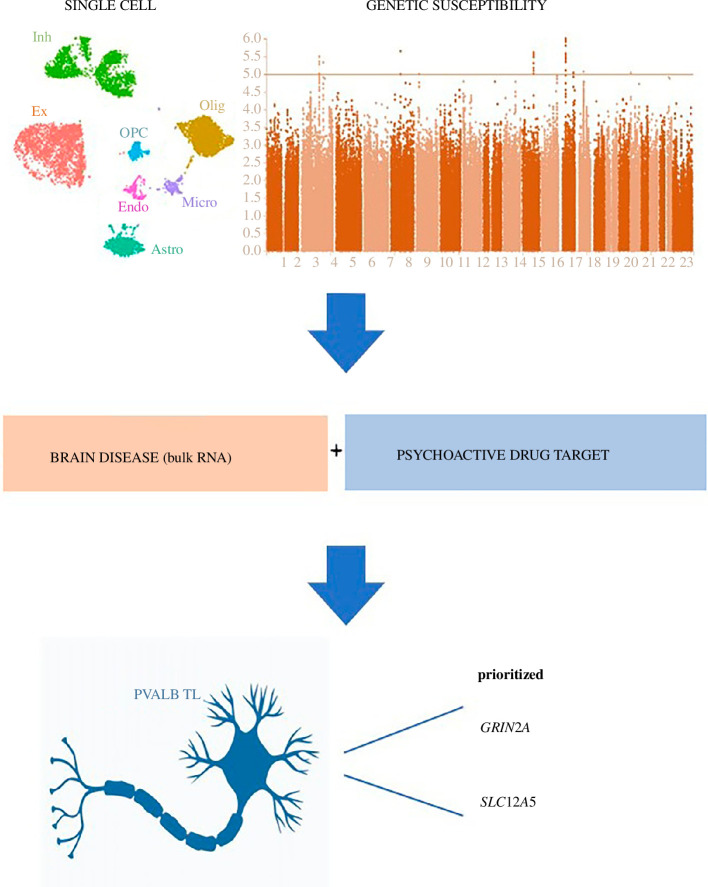
Schematic illustrating the research process for identifying the cell-type vulnerabilities of psychiatric disease and psychoactive drugs.

Psychiatric disorders are complex and challenging to diagnose, with a complex genetic aetiology confirmed by GWAS studies. However, the specific common genetic pathways underlying these disorders are poorly understood [63]. The functional and cell type-specific impact of susceptibility genes in these disorders remains largely unknown [18]. In this study, we constructed a comprehensive single-cell atlas of gene expression changes in the TL and FL using fresh human brain samples. We integrated genetic susceptibility data for seven psychiatric disorders, RNA-seq data from healthy and diseased brain cortex, and drug-specific gene targets (figure 6). Through our integrative analysis, we identified cell type-specific vulnerabilities of disease genes and drug targets, with a specific focus on the PVALB neuronal subtype. These findings emphasize the importance of specific cell types in the genetic basis of psychiatric disorders.

We started from defining the pathways shared between GWAS genes across different neuropsychiatric disorders, such as GABAergic synapse and dopaminergic synapse which modulate several processes relevant to neurodevelopmental disorders, including neuronal proliferation, migration, differentiation and connectivity [64]. Moreover, genetic dysregulation of pathways such as Rap1 and PPAR signalling in the PFC is sufficient to drive stress-relevant cognitive and synaptic phenotypes, and may contribute to inflammatory processes driving neurodegenerative diseases [65,66]. A subset of 38 GWAS genes was common to all psychiatric disorders considered here; this includes genes such as *GRIN2A*, *DGKI* and *SHISA9,* which were previously known to be involved in multiple psychosis [67,68]. These findings suggest that these diverse psychiatric disorders have a genetic relationship with each other, and share genetic pathways and susceptibility genes, suggesting a common genetic basis of these diseases [69].

Integrating our single cell with GWAS genes data helped us understand the cell type context and function of genetic risk factors. The scarcity of reliable animal models for psychiatric diseases also highlights the power of integrative single-cell analysis on freshly obtained human tissue to identify disease-relevant gene expression patterns specific to neuronal subtypes in FL or TL. This cellular-level data can facilitate the development of targeted cell type-specific therapies.

We found that neurons are the most vulnerable cell type to genetic susceptibility, particularly inhibitory neurons. Altered inhibitory control is believed to lead to a change in the power of gamma oscillations [70]. Increase in the ratio between excitation and inhibition, called the E/I balance has been reported in ASD and children with autism show a reduced gamma frequency modulation to a visual task, whereas increased synchrony was observed in ADHD [56,71]. Astrocyte and OPC are also associated with psychiatric disorders, and play essential roles in maintaining brain homeostasis, regulating synaptic transmission and supporting neuronal function. Astrocytes also contribute to maintaining the integrity of the blood-brain barrier and interact closely with neurons. Disruptions in this communication impact neural circuitry, which is relevant to many psychiatric disorders. OPCs generate oligodendrocytes, producing myelin crucial for signal conduction and brain structural integrity, which potentially impacts brain connectivity and communication between brain regions. Among neuronal subtypes, our data suggest that disruption of specific biological processes in PVALB, SST and L5 neurons may contribute to neuropsychiatric disorders. PVALB cells are believed to activate pyramidal neurons only if the signal from EX is sufficient and optimize the signalling in both EX and INH [72]. SST neurons gate excitatory input onto pyramidal neurons within cortical microcircuits, mainly coming from L5 layer of EX which is involved in motor control, decision making and information transfer between the cortex and subcortical structures [73]. These signalling processes, when dysregulated, have been implicated in psychiatric diseases [74]. The relationship between psychiatric disorders and other layers of the cerebral cortex is still under investigation. L2–3 neurons handle local processing, relevant to conditions like schizophrenia and autism. L6 neurons in thalamocortical circuits are crucial for sensory processing and information relay, involving sensory perception abnormalities.

Regional differences between FL and TL are important for psychiatric disorders. The FL is responsible for shaping social behavioural characteristics such as personality, decision making, motivation, while the TL is typically related to emotional regulation, planning, reasoning and problem solving [75]. Here, we annotated regional differences in gene expression at the single-cell level, and tested if these might contribute to explain psychiatric disease susceptibility by GWAS genes. First, our study points to important regional gene expression differences (FL versus TL) in functional pathways affecting both neuronal and subneuronal cell populations. Second, we show GWAS genes for psychiatric diseases are enriched in PVALB cells from the TL, which suggests that shared co-morbidities related to memory deficits might be due, at least in part, to functional dysregulation of PVALB neurons. Memory disorders have been primarily related to destruction in PVALB [76], and defective signal transmission in PVALB neurons has been associated with higher likelihood of developing cognitive deficits [77]. Our main results are supported by analysis of the Allen Brain Atlas dataset, which strengthened our approach for predicting cell types affected by disease. More generally, we report transcriptional changes between FL and TL being related to multiple signalling pathways (e.g. cGMP−PKG, cAMP and long-term potential) and pointed to widespread structural plasticity (of axons, dendrites and synapses). For example, we identified regional gene expression differences impacting synaptic vesicle cycle genes specifically in PVALB neurons (including SNAP-25 and VAMP-2, members of the SNARE complex involved in endocytosis of synaptic vesicle, associate with ADHD) [78]. Our findings are also in keeping with recent work showing a temporal specificity of synaptic change in schizophrenia, with synaptogenesis being predominant earlier in the disease and synaptic loss in chronic phases [79]. Beyond ADHD and BP, a decrease of PVALB inflammatory neurons has been reported in bipolar patients, and this may alter intrinsic inhibitory networks within the superficial layers of the TL, which might disrupt the integration and transfer of information from the cerebral cortex to the hippocampus [80].

We examined the correlation between regional gene expression differences identified in brain tissue from patients without a history of psychiatric disease and gene expression differences in diseased brains or in response to psychoactive drugs. Enrichment for genes dysregulated in the diseased brain and for psychoactive drug-gene targets was the strongest for the inhibitory neuronal subtype, in most cases with specificity to the neurons from TL. DA, BZDs, GABA showed high and similar enrichment in PVALB from TL. Activity of vesicular glutamate transporters, responsible for the uptake of glutamate into the synaptic vesicle [81], is increased in TL in schizophrenia and bipolar patients after treatment with BZDs [82]. There is a link between dopamine D2 receptors and positive symptoms in schizophrenia from TL [83]. Our identification of PVALB neurons as strongly enriched for genes dysregulated in the brain from schizophrenia patients is also in keeping with recent studies in adult macaque cortex sowing schizophrenia DEGs in EX subtypes, where the PVALB-type was the most affected inhibitory subtype [84].

Our integrative analyses identified two key genes, *GRIN2A* (coding subunit 2A of the NMDA-type receptor (NMDAR) and target of DA) and *SLC12A5* (Solute Carrier Family 12 and target of ARA), as being upregulated in PVLAB neurons from TL, upregulated in brain cortex from schizophrenia patients and bipolar patients. Schizophrenic psychosis may originate in the TL due to the deficits in the establishment of synaptic contacts which is caused by PVALB dysfunction, which is mediated by *GRIN2A* in PVALB-positive interneurons [85], and has been proposed for therapeutic targeting. Disruption of *SLC12A5* in PVALB neuron can lead to spontaneous seizures in TL, which help to mediate the electrophysiological effects of GABA [86]. Therefore, by integrating regional gene expression differences at single-cell level with GWAS data, gene dysregulation in disease and response to psychoactive drugs we highlighted *GRIN2A* and *SLC12A5* function in PVALB neurons in the TL.

There are limitations to our approach. First, our study stemmed from the single-cell analysis of only three biological samples. To address these gaps, we compared the DEGs obtained with and without batch correction (electronic supplementary material, figure S15). Importantly, we replicated our main findings through SMART RNA-seq from Allen Brain Atlas. Second, we only included GWAS data for seven psychiatric disorders, and we used a relaxed threshold for the GWAS significance of the genes (GWAS *p*‐value < 10^−5^). The sensitivity of the enrichment analysis was tested by selecting the GWAS genes with various thresholds, which show comparable GWAS enrichment results (electronic supplementary material, figure S14). Moreover, this was used only at the first stage of the analysis to collect a large set of GWAS genes for integrative analyses. The GWAS genes were then filtered (using other datasets) and further refined using more stringent thresholds (GWAS *p*‐value < 10−8) at a later stage of our analysis. Third, we acknowledge that more GWAS studies for schizophrenia and other diseases were published, including careful gene prioritization which are not included in the GWAS catalogue. Therefore, we compared our main findings (enrichment and the transcriptome differences between TL and FL) with a recently prioritized gene list for schizophrenia [87] which showed an overlap with our main findings (electronic supplementary material, figure S17). Fourth, we used the overrepresentation-based hypergeometric test for enrichment analysis, which has limitations. We also relied on the ‘proximity based’ GWAS SNP-to-gene mapping provided by the NHGRI-EBI GWAS DB. Overrepresentation-based tests typically rely on defining two groups of genes (DEGs and non-DEGs) based on a *p*-value cut-off for the significance of the DEGs. While other enrichment methods that improve on SNP-to-gene mapping are available (e.g. MAGMA and LDSC) [14,88], these require access to the whole SNP associations; here we used GWAS SNP data (*p* ≤ 10^−5^) available in the NHGRI-EBI GWAS DB, and overrepresentation-based approaches that have been successfully employed before [30,31].

In summary, we generated a single-cell-resolved dataset from TL and FL tissues, which provided new insights into the regional brain differences and cell type-specific vulnerabilities to neuropsychiatric disorders and drugs. Recent studies investigated the cell type vulnerability to psychoactive drugs [18], highlighting the role or SST and VIP interneurons. Our study identified additional sets of neurons (PVALB and LAMP5) as highly vulnerable cells to psychoactive drugs and show this vulnerability is specific to neurons from the TL. Integrating brain single cell with GWAS data, Olislagers *et al*. [89] showed that neuronal cell subsets are consistently implicated in several psychiatric disorders. Our study confirms and extends these results, as we further refined the neuronal subtypes to the inhibitory subtype (PVALB and SST) and again highlight their specificity to the TL. However, differently from [18] and [89], in our study, we jointly integrated multiple datasets: regional single-cell analysis in TL/FL, GWAS data, gene expression (bulk RNA) in brain from psychiatric disorders patients and gene response to psychoactive drugs. This allowed us to disentangle the relative contribution of genetic susceptibility and psychoactive drugs to the cell type vulnerability. Overall, of the genes upregulated in PVALB from TL, we found 9.8% being enriched for GWAS genes and 35.6% being enriched for psychoactive drug targets, respectively. This suggests a considerably larger contribution (2.1-fold) of psychoactive drugs to PVALB cell vulnerability in the TL. Finally, we provide a unique data resource with over 45 000 cells and high sequencing depth (median UMI per cell = 825, UMI of 1/3 cells is over 1000) [90] and present a detailed integrative single-cell analysis of cellular heterogeneity and brain regional changes of psychiatric disease genes, which will enable specific investigations of disease risk genes and their cell type-specific contribution to disease susceptibility.

## Data Availability

We have made the raw data and metadata available at Zenodo [91]. Supplementary material is available online [92].
